# Early neurotrophic pharmacotherapy rescues developmental delay and Alzheimer’s-like memory deficits in the Ts65Dn mouse model of Down syndrome

**DOI:** 10.1038/srep45561

**Published:** 2017-04-03

**Authors:** Syed Faraz Kazim, Julie Blanchard, Riccardo Bianchi, Khalid Iqbal

**Affiliations:** 1 Department of Neurochemistry, and SUNY Downstate/NYSIBR Center for Developmental Neuroscience, New York State Institute for Basic Research (NYSIBR), Staten Island, NY 10314, USA; 2 The Robert F. Furchgott Center for Neural and Behavioral Science, and Department of Physiology and Pharmacology, State University of New York (SUNY) Downstate Medical Center, Brooklyn, NY 11203, USA; 3 Graduate Program in Neural and Behavioral Science, SUNY Downstate Medical Center, Brooklyn, NY 11203, USA

## Abstract

Down syndrome (DS), caused by trisomy 21, is the most common genetic cause of intellectual disability and is associated with a greatly increased risk of early-onset Alzheimer’s disease (AD). The Ts65Dn mouse model of DS exhibits several key features of the disease including developmental delay and AD-like cognitive impairment. Accumulating evidence suggests that impairments in early brain development caused by trisomy 21 contribute significantly to memory deficits in adult life in DS. Prenatal genetic testing to diagnose DS *in utero*, provides the novel opportunity to initiate early pharmacological treatment to target this critical period of brain development. Here, we report that prenatal to early postnatal treatment with a ciliary neurotrophic factor (CNTF) small-molecule peptide mimetic, Peptide 021 (P021), rescued developmental delay in pups and AD-like hippocampus-dependent memory impairments in adult life in Ts65Dn mice. Furthermore, this treatment prevented pre-synaptic protein deficit, decreased glycogen synthase kinase-3beta (GSK3β) activity, and increased levels of synaptic plasticity markers including brain derived neurotrophic factor (BNDF) and phosphorylated CREB, both in young (3-week-old) and adult (~ 7-month-old) Ts65Dn mice. These findings provide novel evidence that providing neurotrophic support during early brain development can prevent developmental delay and AD-like memory impairments in a DS mouse model.

Down syndrome (DS), caused by triplication of human chromosome 21 (Hsa21, trisomy 21), is the most common genetic cause of intellectual disability and is associated with a greatly increased risk of early-onset Alzheimer’s disease (AD)^1^,^2^. By the age 40, almost all DS individuals develop the histopathological characteristics of AD including amyloid plaques and neurofibrillary tangles^3^,^4^,^5^,^6^, and ~70% of individuals with DS develop dementia by age 60^2^,^7^,^8^. Currently, there are ~250,000–400,000 individuals with DS in the United States and approximately five million worldwide, and as yet, there is no effective pharmacological treatment that can either rescue the neurodevelopmental and intellectual deficits or reduce the risk of AD in adult life in DS^9^,^10^,^11^,^12^.

During the last few decades, several mouse models of DS with segmental trisomy for orthologues of Hsa21 genes have been developed to facilitate our understanding of the link between enhanced gene dosage and DS phenotype such as developmental delay and altered synaptic plasticity and cognition^13^,^14^,^15^. The Ts65Dn mouse, the most widely used mouse model of DS, is trisomic for the distal portion of mouse chromosome 16 (MMU16) which contains more than 140 genes orthologous to those on Hsa21^16^,^17^,^18^,^19^,^20^. In the newborn period, Ts65Dn mice mimic the human condition presenting developmental delay in motor and sensory milestones^18^,^21^. Like human DS individuals, these mice demonstrate impaired ontogenic neurogenesis^22^, possibly a major factor responsible for aberrant brain development and cognitive dysfunction. The adult Ts65Dn mice show deficient adult hippocampal neurogenesis^23^,^24^, suppressed synaptic plasticity (long-term potentiation, LTP)^25^,^26^,^27^, and impairment in spatial and declarative memory^28^,^29^,^30^,^31^,^32^,^33^,^34^,^35^,^36^.

Most of the pharmacological approaches used in DS mouse models and human DS patients to ameliorate cognitive and other neurological deficits have targeted the adult or adolescent life stages^9^,^10^,^11^,^37^,^38^,^39^. Prenatal genetic testing, including amniocentesis and chorionic villous sampling (CVS) to diagnose DS in early *in utero* period, provides the novel opportunity to initiate pharmacological treatment which can positively affect early brain development and significantly improve the postnatal cognitive outcome in DS^40^,^41^,^42^,^43^. During the past few years, a growing number of studies have explored the exciting possibility of using pharmacological approaches to ameliorate neurodevelopmental deficits in DS mouse models during the critical time window of brain development, and shown significant improvement in learning and memory in adult life (for review, refs 40, 41, 42 and 44).

It is worthwhile to note that even though studies carried out in adult DS mice have shown that it is possible to improve hippocampus-dependent learning and memory, the duration of this beneficial effect in most cases remains controversial^42^. After the critical window of brain development including neurogenesis and synaptogenesis which takes place during prenatal and early postnatal period, the brain can undergo limited plastic changes, and the late therapies are unlikely to exert profound and long lasting effects in DS brains^40^,^41^,^42^,^44^. Thus, early therapies could hold the key to rescue Alzheimer’s like cognitive impairment in DS individuals.

During early brain development, neurotrophic factors provide the appropriate brain milieu necessary for all aspects of neural development, including neuronal proliferation, differentiation, growth, and migration^45^,^46^,^47^,^48^. The trisomy 21 in DS, which leads to overexpression of nearly 300 genes, may alter the trophic support provided by neurotrophic factors during early brain development^3^,^43^,^49^. For instance, increased amyloid β precursor protein (APP) gene dosage has been linked to failed nerve growth factor (NGF) signaling and cholinergic neurodegeneration in Ts65Dn mice^50^. Similarly, increased superoxide dismutase 1 (SOD1) gene dosage is known to cause increased oxidative stress in DS^51^; increased oxidative stress has previously been shown to block ciliary neurotrophic factor (CNTF) activity in neurons, which is essential for neuronal survival and maintenance^52^,^53^,^54^. The expression of brain derived neurotrophic factor (BDNF), which plays essential role in neurodevelopment, has been shown to be decreased in the hippocampus of postnatal day 15 (PND15) Ts65Dn mice^22^. An inverse correlation between dual-specificity tyrosine phosphorylation-regulated kinase 1A (DYRK1A) overexpression because of trisomy 21 and reduced BDNF expression has been suggested^55^. Counteracting the trisomy 21 induced alteration in neurotrophic support which is essential for early brain development is a novel therapeutic modality for DS. Adjusting the brain milieu by modulating neurotrophic support at the critical period of fetal brain development may ameliorate neurodevelopmental abnormalities and cognitive impairment associated with DS.

Neurotrophic factors, by virtue of their neurogenic and neurotrophic activities, have potential for treatment of cognitive impairment in DS and AD^56^,^57^,^58^,^59^. However, the therapeutic usage of neurotrophic factors such as BDNF and CNTF is hindered by limited blood-brain barrier (BBB) permeability, poor plasma stability and unfavorable pharmacokinetics, and systemic adverse effects^56^,^60^,^61^,^62^. Neurotrophic factor small-molecule mimetics, which can exert the therapeutic beneficial effects of neurotrophic factors on neurogenesis, neuronal and synaptic plasticity, and cognition, and have favorable pharmacokinetics and BBB permeability without unwanted systemic effects, offer a promising strategy to overcome the limitations associated with therapeutic usage of whole-molecule recombinant neurotrophic factors^63^,^64^,^65^.

Previously, by employing neutralizing antibodies and epitope mapping, our laboratory devised a CNTF small-molecule peptide mimetic, Peptide 021 (P021; Fig. 1a) which was found to ameliorate learning and memory deficits in animal models of aging and AD^66^,^67^,^68^,^69^. The compound P021 exerts a neurogenic and neurotrophic effect by inhibiting leukemia inhibitory factor (LIF) signaling pathway and enhancing brain derived neurotrophic factor (BDNF) expression by increasing its transcription^66^,^67^,^69^,^70^,^71^,^72^,^73^,^74^,^75^. P021 was shown to ameliorate AD pathology via reduction in glycogen synthase kinase -3 beta (GSK3β) activity in a triple transgenic mouse model of AD (3xTg-AD mouse)^67^. P021 is a small water-soluble compound that is BBB-permeable and has suitable pharmacokinetics for oral administration without any adverse effects observed with native CNTF or BDNF molecule^66^,^67^,^69^.

In the present study, we treated Ts65Dn mice from embryonic day 8 (E8) to weaning/postnatal day 21 (PND21) with the compound P021 (200 nmoles/g diet administered to mothers/dams). We report that treating the Ts65Dn mice with a neurotrophic factor small-molecule peptide mimetic during prenatal and early postnatal period can ameliorate the developmental delay in early postnatal period and AD-like learning and memory impairments in adult life.

## Methods

### Study outline

**Figure 1:**
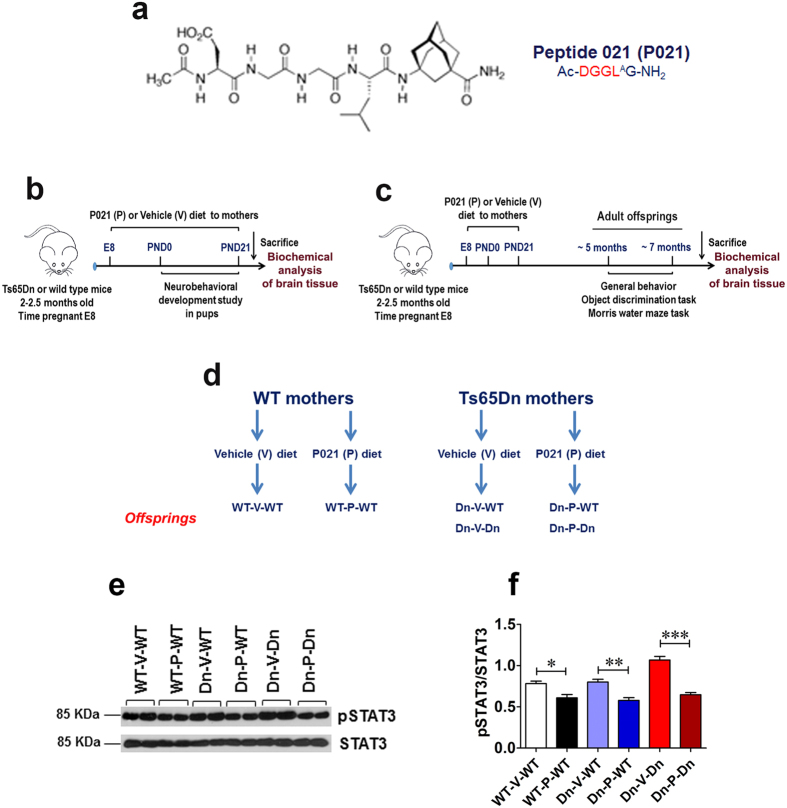
Structure of compound P021, study design and groups, and placental/lactational and BBB permeability of P021. (**a**) Structure of CNTF derived P021 (Ac-DGGL^A^G-NH_2_; molecular weight, 578.3). (**b**) Design of the neurobehavioral development component of the study. The effect of prenatal to early postnatal treatment on the appearance of developmental milestones/early neurological reflexes was evaluated from PND1 to PND21. (**c**) Design of the longitudinal component of the study. The effect of prenatal to early postnatal treatment on cognition and general behavior in adult mice was evaluated. (**d**) Summary of the study groups. There were 6 study groups as follows: WT offsprings of WT mothers on vehicle diet (WT-V-WT), WT offsprings of WT mothers on P021 diet (WT-P-WT), WT offsprings of Ts65Dn mothers on vehicle diet (Dn-V-WT), WT offsprings of Ts65Dn mothers on P021 diet (Dn-P-WT), Ts65Dn offsprings of Ts65Dn mothers on vehicle diet (Dn-V-Dn), and Ts65Dn offsprings of Ts65Dn mothers on P021 diet (Dn-P-Dn). (**e**,**f**) Based on previous studies on the mechanism of action of compound P021^67^,^69^, the delivery of P021 to the target organ, i.e. offspring’s brain via placental barrier and during lactation, and across BBB, was determined by evaluating the phosphorylation level of STAT3 at Tyr705 in the hippocampus of 3-week-old WT and Ts65Dn mice. P021 treatment significantly reduced phosphorylation of STAT3 at Tyr705 in the hippocampal tissue of both WT and Ts65Dn mice. This confirmed that significant amount of P021 reached the offsprings’ brains to exert its proposed effect i.e. inhibition of LIF activity as measured by pSTAT3, Tyr705. Quantification of the Western blots is shown as mean ± S.E.M. from WT-V-WT (n = 7), WT-P-WT (n = 6), Dn-V-WT (n = 7), Dn-P-WT (n = 7), Dn-V-Dn (n = 7), and Dn-P-Dn (n = 7) mice. *p < 0.05; **p < 0.01; ***p < 0.001. One-way ANOVA with Bonferroni’s *post*-*hoc* test.

We treated 2–3-month-old pregnant Ts65Dn (Dn) and wild-type (WT) mothers from day 8 (E8) of pregnancy till the weaning of the pups on PND21 with compound P021 (P) in the feed, or as control with vehicle (V) feed (Fig. 1). The offsprings also had access to the P021 or vehicle diet as per mother’s group till PND21. The offsprings from Ts65Dn mothers were genotyped to identify Ts65Dn and WT pups. There were 6 study groups as follows: WT offsprings of WT mothers on vehicle diet (WT-V-WT), WT offsprings of WT mothers on P021 diet (WT-P-WT), WT offsprings of Ts65Dn mothers on vehicle diet (Dn-V-WT), WT offsprings of Ts65Dn mothers on P021 diet (Dn-P-WT), Ts65Dn offsprings of Ts65Dn mothers on vehicle diet (Dn-V-Dn), and Ts65Dn offsprings of Ts65Dn mothers on P021 diet (Dn-P-Dn). In the first phase of the study (Fig. 1b), the newborn pups were evaluated for neurobehavioral development starting on PND1 and carried out until PND21 (or until the appearance of developmental milestone/reflex). After completion of behavioral testing, the 3-week-old mice were sacrificed and brain tissue was collected. The neurobehavioral development component of the study was performed on WT-V-WT (n = 11), WT-P-WT (n = 10), Dn-V-WT (n = 10), Dn-P-WT (n = 9), Dn-V-Dn (n = 11), and Dn-P-Dn (n = 12) mice. In the second phase of the study (Fig. 1c), after prenatal to early postnatal treatment, the offsprings were allowed to grow till 5 months of age, and then general behavioral evaluation and cognitive testing were performed. After weaning, the offsprings received regular mouse chow. After completion of behavioral testing, ~7-month-old animals were sacrificed and brain tissue was collected for biochemical analyses. This longitudinal component of the study was carried out on WT-V-WT (n = 10), WT-P-WT (n = 12), Dn-V-WT (n = 12), Dn-P-WT (n = 11), Dn-V-Dn (n = 13), and Dn-P-Dn (n = 12) mice. Data was pooled together for male and female mice as no statistically significant gender differences were noted in separate analysis. The study groups are summarized in Fig. 1d.

### Design and synthesis of P021

The peptidergic compound P021 (Ac-DGGL^A^G-NH_2_; molecular weight: 578.3) corresponds to a biologically active region of human CNTF molecule (amino acid residues 148–151) to which adamantylated glycine was added to make it more stable and lipophilic^66^,^67^,^68^,^69^,^71^,^75^. The peptide was synthesized and purified by reverse phase high performance liquid chromatography (HPLC) to >96% purity, as described previously^69^. The sequence of the peptide was confirmed by mass spectrometry.

### Stability in plasma and gastric and intestinal juices, blood-brain barrier permeability, and placental/lactational accessibility of P021

The studies regarding the plasma stability and stability in gastric and intestinal juices of P021 were done at EVER NeuroPharma GmbH, Unterach, Austria^67^. Briefly, the compound P021 was found stable both in artificial gastric juice (>90%) and in artificial intestinal juice (>95%) as analyzed up to 30 min and 2 hours, respectively. P021 was found to be BBB-permeable based on studies in adult C57Bl/6 mice carried out through a commercial service (APREDICA, Watertown, MA, USA) and described previously^67^. In a previous study, we found that P021 acted via dose-dependent competitive inhibition of LIF activity towards LIF/CNTF receptor at as low as 0.1 nM (*p* < 0.05 at 10 nM P021) concentration as measured by phosphorylation of signal transducer and activator of transcription 3 (STAT3) at Tyr705^69^. Also, previously we showed that oral administration of P021 in the diet decreased the phosphorylation of STAT3 at pTyr705 in the hippocampus of 3xTg-AD mice and the age-matched wild type controls after 6 months of treatment^67^. These data provided further evidence that the compound P021 is BBB permeable and is orally bioavailable. In the present study, we found that prenatal to early postnatal treatment with P021 also resulted in a significant reduction in phosphorylation of STAT3 at Tyr705 in the hippocampus of 3-week-old WT and Ts65Dn mice (Fig. 1e,f). These data suggest that P021 was delivered across placental barrier during pregnancy and via breast milk during lactation, and then via BBB to reach significant concentrations in off springs’ brains to inhibit STAT3 phosphorylation. It is also worth mentioning that young mice may have consumed some P021 diet directly as young mice start consuming solid food by ~PND16.

### Animals and housing

Female Ts65Dn [C57Bl/6JEiJxC3Sn.BLiA-Ts(17^16^)65Dn/DnJ; Stock no. 005252] mice, carrying a partial trisomy of chromosome 16^18^,^76^, were purchased from Jackson Laboratories (New Harbor, ME, USA) and maintained keeping the original genetic background by mating them with C57BL/6JEiJxC3Sn.BLiAFi (Stock no. 003647) males. As compared to earlier strains, this genetic background is homozygous for the WT allele of *Pde6b* and thus all the trisomic mice produced can be used for studies without concern for retinal degeneration^29^. Mice were housed and bred in accordance with approved protocols from the Institutional Animal Care and Use Committee (IACUC) of New York State Institute for Basic Research (NYSIBR), in accordance with the PHS Policy on Human Care and Use of Laboratory Animals (Protocol numbers 199 and 383). Control animals (WT groups) were generated by mating non-trisomic (2N) littermate mice. Animals were group-housed (4 animals per cage) with a 12:12-hour light dark cycle and with *ad libitum* access to food and water.

### Genotyping of animals

The Ts65Dn mice and their diploid littermates (2N) were genotyped using a real-time quantitative polymerase chain reaction (RT-qPCR) analysis^77^. The tail or toe clippings were obtained from 10-day-old mouse pups and were stored at −20 °C till further processing. The genomic DNA was extracted from clippings using Wizard Genomic DNA Purification Kit per manufacturer’s instructions (Promega, Madison, WI, USA). The RT-qPCR was done using Brilliant SYBR Green Master Mix (Agilent, Santa Clara, CA, USA) in a Stratagene Mc3000p PCR detection system under the following conditions: 10 min at 95 °C, 40 cycles of denaturation at 95 °C for 30 s, annealing 55 °C for 1 min, extension at 72 °C for 1 min. The fold change in APP gene (3 copies in Ts65Dn mice) was evaluated. The apolipoprotein b (apob) gene (2 copies in Ts65Dn mice) was used as internal control. The primer sequences were the following: forward 5′-TGCTGAAGATGTGGGTTCGA-3′ and reverse 5′-GACAATCACGGTTGCTATGACAA-3′ for APP; forward 5′-CACGTGGGCTCCAGCATT-3′ and reverse 5′-TCACCAGTCATTTCTGCCTTTG-3′ for apob. The genotype was determined using ΔΔCT method by comparing ΔCT value of each unknown sample against a known calibrator average ΔCT^77^.

### Treatment of animals with P021

The 2-3-month-old time pregnant Ts65Dn (n = 35) and WT control (n = 20) mice were treated orally from day 8 (E8) of pregnancy till the new born pups were 21 days old (PND21) with P021 (P) or vehicle (V) diet for a total of 5-week period. Treatment was administered as 200 nM P021/g formulated diet (Research Diets, New Brunswick, NJ, USA) with *ad libitum* access to mothers/dams. The dosage of P021 in diet was determined based on our previous study on P021 treatment in 3xTg-AD mice^67^. The vehicle-treated control animals received the same diet but without the compound. For the longitudinal study, the offsprings received regular mouse chow after PND21.

### Behavioral procedures

#### General examination

For both the neurobehavioral development in mouse pups and the longitudinal adult mice studies, the physical state and condition of the animals (both mothers and offsprings) were carefully examined throughout the respective study duration. Body weight of mouse pups was recorded daily from PND1 to PND21. Body weight of adult mice was evaluated monthly. Adult mice were not subjected to any behavioral testing till they were ~5 months of age.

#### Neurobehavioral development

Evaluation of neurobehavioral development in rodents represents an essential study paradigm to model neurodevelopmental disorders such as Down syndrome and autism spectrum disorders (ASDs) which are characterized by mental retardation and delays in growth and development^78^,^79^,^80^. In rodents like mice and rats, the early postnatal period is associated with a burst of brain growth, synaptogenesis, myelination, and development of motor and sensory modalities^78^,^79^. Therefore, examination of neurobehavioral development in rodents allows to trace the nervous system ontogeny via evaluation of neurological reflexes, developmental milestones, and early motor behavior including muscle power and coordination^79^.

In the present study, evaluation of neurobehavioral development was performed following the procedure described previously by others^21^,^79^,^81^, and further validated by us ref. 80. Examination was started on PND1 and was carried out until PND21 (or until the appearance of developmental milestone/neurological reflex) daily between 12:00–15:00 hours in a set up made specifically for the purpose in the Behavior Lab of the Department of Neurochemistry at NYSIBR. The following neurological reflexes and developmental milestones were examined by the same evaluators blinded to both the genotype and the treatment group.

##### Surface righting

The mouse pup was put on its back with the experimenter’s fingers holding the head and the hind body. The time taken in seconds to turn over with all four paws placed on the surface of the table after release was measured. Surface righting was evaluated daily until the pup could right itself in <5 s for two consecutive days. The data for the day of first appearance of surface righting were analyzed. The surface righting reflex measures labyrinthine and body righting mechanisms, and motor strength and coordination^79^.

##### Negative geotaxis

The mouse pup was placed head down on a square of screen inclined at an angle of 45°. The time taken by the pup to turn around 180° to the head up position was evaluated. The test was halted if the pup did not turn around within 30 s. If the mouse pup lost grip and slipped, it was again placed at the start point once. The negative geotaxis test was repeated daily until the mouse pup could perform appropriately in less than 30 s for two consecutive days. The data for the first day of appearance of negative geotaxis were analyzed. Like surface righting, the negative geotaxis reflex also measures labyrinthine and body righting mechanisms, and motor strength and coordination^79^.

##### Cliff aversion

The mouse pup was placed on cliff edge (eppendorf box) with the snout and fore limbs over the edge, and the time taken (in seconds) to turn and move away was recorded. The test was repeated every day until the mouse pup could perform appropriately in less than 30 s for two consecutive days. The first day of appearance of cliff aversion data were analyzed. The cliff aversion test measures labyrinthine function, motor strength and coordination, and feel sensitivity^21^,^79^.

##### Rooting reflex

A cotton swab was rubbed from front to back along the side of the head and snout, and the head turning response towards this tactile stimulus was observed. The rooting reflex was evaluated daily till the pup responded correctly for 2 consecutive days. Rooting is a measure of tactile sensory reflex and motor coordination^21^,^79^.

##### Forelimb grasp

The mouse pup was held with its forepaws grasping a string fixed from one end to the other end of the cage nearly 12 cm above the bedding. The pup was released, and the time the pup spent holding onto the string with forepaws was recorded. The test was considered positive when the pup held onto the string for >2 s. It was repeated daily until performed correctly for 2 consecutive days. Forelimb grasp testing provides a measure of motor strength^79^.

##### Air righting

The mouse pup was held upside for few seconds approximately 12 cm above the soft bedding of a cage and was then released. The test was marked positive on the day when the pup landed with all its fore and hind paws placed on the surface of the bedding. It was repeated every day until positive for 2 consecutive days. Similar to surface righting, air righting measures labyrinthine and body righting mechanisms, and motor coordination^79^.

##### Eye opening

The mouse pups were inspected daily for first day of opening of both eyes. Eye opening is a developmental milestone.

##### Ear twitch reflex

The pulled-out end of a cotton swab was applied against the ear tip, and the presence or absence of flattening of the ear against the side of the head was observed. The ear twitch reflex was evaluated daily until the mouse pup responded appropriately for 2 consecutive days. It is a measure of sensory tactile reflex^79^.

##### Auditory startle

To evaluate auditory startle, the experimenter clapped within 10 cm of the mouse pup, and the first day of the startle response was recorded. It was repeated daily until positive for 2 consecutive days. Auditory startle evaluates auditory reflex^79^,^81^.

### Elevated plus maze

Elevated plus maze testing was used to evaluate the anxiety-like behavior^82^ in adult mice. The maze comprised of four arms (30 × 5 cm) connected by a common 5 × 5 cm center area. There were two opposite facing open arms (OA), and two facing closed arms (CA) which were enclosed by 20 cm-high walls. The entire plus-maze was elevated on a wooden pedestal to a height of 82 cm above the floor level in a room separated from the experimenter. Ambient luminosity was kept at 60 lux to control the anxiogenic feature of light for mice. Each mouse was placed onto the central area facing an open arm and allowed to explore the maze for a single 8 min session. After each session, any feces were removed from the maze, and the floor of the maze was wiped with 70% alcohol to remove any urine or scent cues. The number of entries into OA and CA, and the amount of time spent in OA and CA were recorded by a video tracking system (ANY-Maze software, version 4.5, Stoelting Co., Wood Dale, IL, USA). As OA are more anxiogenic for rodents than CA, the percentage of time spent in OA [OA/(OA + CA) × 100] was calculated to evaluate anxiety-like behavior of mice.

### Open field

We evaluated the exploratory activity in adult mice by allowing them to freely explore the open field arena in a single 15-min-session. The testing apparatus was a classic open field i.e., a PVC square arena with dimensions of 50 × 50 cm and 40 cm high walls, as described previously^67^,^72^. The open field was placed in a room separated from the experimenter, and was surmounted by a video camera connected to a computer tracking mice. Ambient luminosity in the open field was maintained at 60 lux that is believed to be non-anxiogenic for rodents. Data were recorded using a video tracking system (ANY-Maze software, version 4.5, Stoelting Co., Wood Dale, IL, USA). The parameters analyzed included ambulatory distance and time during the single 15-min-session.

### One-trial object recognition/discrimination task

We evaluated the long-term memory performance of the adult mice in a one-trial object recognition/discrimination task in an open field arena. The test is based on the innate tendency of rodents to differentially explore novel objects over familiar ones, as described before^83^,^84^,^85^. The task protocol consisted of three different phases: a habituation phase (four sessions of 10 min each on four consecutive days), a sample phase (fifth day), and a test phase (sixth day). During the first four days, the study mice were individually subjected each day to a habituation session of 10 min during which they were introduced in the empty open field arena to familiarize them with the arena. On the fifth day, each mouse was first subjected to the sample phase where two identical cylindrical plastic objects were placed in a symmetric position from the center of the arena, and the mouse was allowed to freely explore the objects for 10 min. The exploration of the objects was defined as any investigative behavior such as head orientation or sniffing, or contact that occurred with an object in a distance <2 cm. On the sixth day (retention period, 24 h), each mouse was introduced into the arena to do the test phase. During the test phase, the mouse was exposed to two objects for another 10 min: a familiar object (previously presented during the sample phase; a cylindrical plastic object) and a new object (a triangular plastic object), placed at the same location as that of the other object during the sample phase. The data was collected using a video tracking system (ANY-Maze software, version 4.5, Stoelting Co., Wood Dale, IL, USA). Object discrimination (measure of novelty preference) was evaluated by applying the following index to the test phase data:


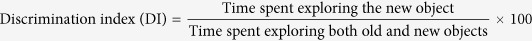


### Spatial reference memory task in the Morris water maze

Spatial reference learning and memory were tested in the Morris water maze (MWM) task adapted from that originally described by Morris and collaborators^86^, and validated in AD mice by us previously^67^,^72^,^87^. Mice were trained for 4 trials (90 s each) per day and for a total of 4 consecutive days in a circular water tank (diameter, 180 cm) filled with water (at room temperature, 21 + 2°C) rendered opaque with small amount of non-toxic white paint. Four positions around the edge of the tank were designated for alternative start positions for successive trials. The tank was surrounded by extra-maze cues in the visual discrimination range detectable by mice. During the training trials, a circular platform (diameter, 14 cm) was submerged 1 cm below water surface in the target quadrant (TQ). If a mouse was unable to locate the platform within 90 s of a training trial, it was gently guided to it. At the end of each trial, the mouse was left on the platform for 20 s, then dried, and returned to its home cage until the next trial. With 4 trials/day for 4 consecutive days, each mouse performed a total of 16 training trials. Probe trial for memory was given 24 h after the last day of training. For the probe trial, the circular tank was divided into 4 imaginary quadrants (TQ; AR, adjacent right; AL, adjacent left; OQ, opposite quadrant). During the probe trial, mice were allowed to swim in the pool without escape platform for 60 s. The latency to reach the platform during training trials and the time spent in the 4 quadrants during probe trial was recorded using an automated video tracking system with SMART (Panlab, San Diego Instruments) v 2.0.14 software.

### Tissue processing

After completion of behavioral testing, the 3-week-old mice from the neurobehavioral development study and the ~7-month-old mice from the longitudinal study were anesthetized with an overdose of sodium pentobarbital (125 mg/kg) and were transcardially perfused with 0.1 M phosphate buffered saline (PBS). The mouse brains were extracted immediately after perfusion. The left hemisphere of the brain was dissected into hippocampus, cerebral cortex, cerebellum, and brain stem, immediately frozen on dry ice, and then stored at −80 °C till further processing for biochemical analysis.

### Quantitative real time polymerase chain reaction (RT-*q*PCR) analysis

The total RNA was extracted from a small piece of left cerebral cortex using RNeasy plus mini kit (Qiagen, Valencia, CA, USA) as per manufacturer’s instructions. Complementary DNA synthesis was performed by using SuperScript first strand kit (Invitrogen, Carlsbad, CA, USA). The RT-qPCR was carried out using Brilliant SYBR Green Master Mix (Agilent, Santa Clara, CA, USA) in a Stratagene Mc3000p PCR detection system employing the following conditions: 10 min at 95 °C, 40 cycles of denaturation at 95 °C for 30 s, annealing 55 °C for 1 min, extension at 72 °C for 1 min. The primer sequences were the following: forward 5′-CCGCCAGACAGGAAACACAT-3′ and reverse 5′-AACCCAGAGCACCAGGTTCA-3′ for the synaptophysin; forward 5′-GATGAGGACCAGAAGGTTGG-3′ and reverse 5′-GATTGGGTAGTTGGGCATTG-3′ for BDNF; forward 5′-GGTCCTCAGACTGGCCTACA-3′ and reverse 5′-GCTCCTGGTCCTGTCAACTC-3′ for BDNF receptor, tropomyosin receptor kinase B (TrkB); forward 5′- CTGATTCCCAAAAACGAAGG-3′ and reverse 5′-CTGCCCACTGCTAGTTTGGT-3′ for cAMP response element-binding protein (CREB); and forward 5′-AGGTCGGTGTGAACGGATTTG-3′ and reverse 5′-TGTAGACCATGTAGTTGAGGTCA-3′ for glyceraldehyde 3-phosphate dehydrogenase (GAPDH). Relative quantification was performed using the ΔΔCt method.

### Western blots

The hippocampus from left cerebral hemisphere stored at −80 °C from each mouse was homogenized in a Teflon-glass homogenizer to obtain 10% (w/v) homogenate. The pre-chilled homogenization buffer comprised of 50 mM Tris–HCl (pH 7.4), 8.5% sucrose, 2 mM EDTA, 2 mM EGTA, 10 mM β-mercaptoethanol plus the following protease and phosphatase inhibitors: 0.5 mM AEBSF, 10 μg/mL aprotinin, 10 μg/mL leupeptin, 4 μg/mL pepstatin, 5 mM benzamidine, 20 mM β-glycerophosphate, 50 mM sodium fluoride, 1 mM sodium orthovanadate, and 100 nM okadaic acid. Modified Lowry assay was used to calculate protein concentration of each brain homogenate^88^. The tissue homogenates were boiled in Laemmli’s buffer for 5 min, and then subjected to 10% or 12% SDS-polyacrylamide gel electrophoresis followed by transfer of separated proteins on 0.45 μm Immobilon-P membrane (Millipore, Bedford, MA, USA). The following primary antibodies were used: mouse monoclonal anti-synaptophysin (1:3000, Millipore, Temecula, CA, USA), rabbit monoclonal anti-PSD95 (1:1000, Cell Signaling Technology, Danvers, MA, USA), rabbit polyclonal anti-BDNF, N-20 (1:500, Santa Cruz Biotechnology, Santa Cruz, CA, USA), rabbit polyclonal anti-CNTF, FL-200 (1:500, Santa Cruz Biotechnology, Santa Cruz, CA, USA), rabbit polyclonal anti-TrkB (total) (1∶500; Santa Cruz Biotechnology, Santa Cruz, CA, USA), rabbit polyclonal anti-CREB (1:1000, Cell Signaling Technology, Danvers, MA, USA), rabbit polyclonal anti-phosphorylated CREB, Ser133 (1:1000, Cell Signaling Technology, Danvers, MA, USA), rabbit monoclonal anti GSK3β (1:1000, Cell Signaling, Danvers, MA, USA), rabbit polyclonal anti-phosphorylated GSK3β, Ser9 (1:1000, Cell Signaling, Danvers, MA, USA), rabbit polyclonal anti-protein kinase A catalytic subunit alpha (PKAcα) (1:1000, Santa Cruz Biotechnology, Santa Cruz, CA, USA), mouse monoclonal anti-STAT3 (1:1000; Cell Signaling Technology, Danvers, MA, USA), rabbit monoclonal anti-pSTAT3, Tyr705 (1:1000; Cell Signaling Technology, Danvers, MA, USA), and rabbit polyclonal antibody to GAPDH (1:1000, Santa Cruz Biotechnology, Santa Cruz, CA, USA) as loading control. The blots were developed and quantified as described before^67^,^80^. For quantification of different protein levels, each immunoreactive band was normalized to its corresponding GAPDH band.

### Statistical analysis

The statistical analyses were performed using GraphPad Prism version 6.0 (GraphPad Software Inc., La Jolla, CA, USA). Data are presented as mean ± S.E.M. The Kolmogorov–Smirnov test was applied to determine the normality of the data. The one-way or two-way ANOVA followed by *post hoc* Bonferroni’s test was used for analysis involving multiple groups. Grubb’s test was employed to identify the statistically significant outliers in all data sets. For all purposes, *p* < 0.05 was considered as statistically significant.

## Results

### Prenatal to early postnatal treatment with P021 rescues delay in neurobehavioral development in Ts65Dn pups

**Figure 2:**
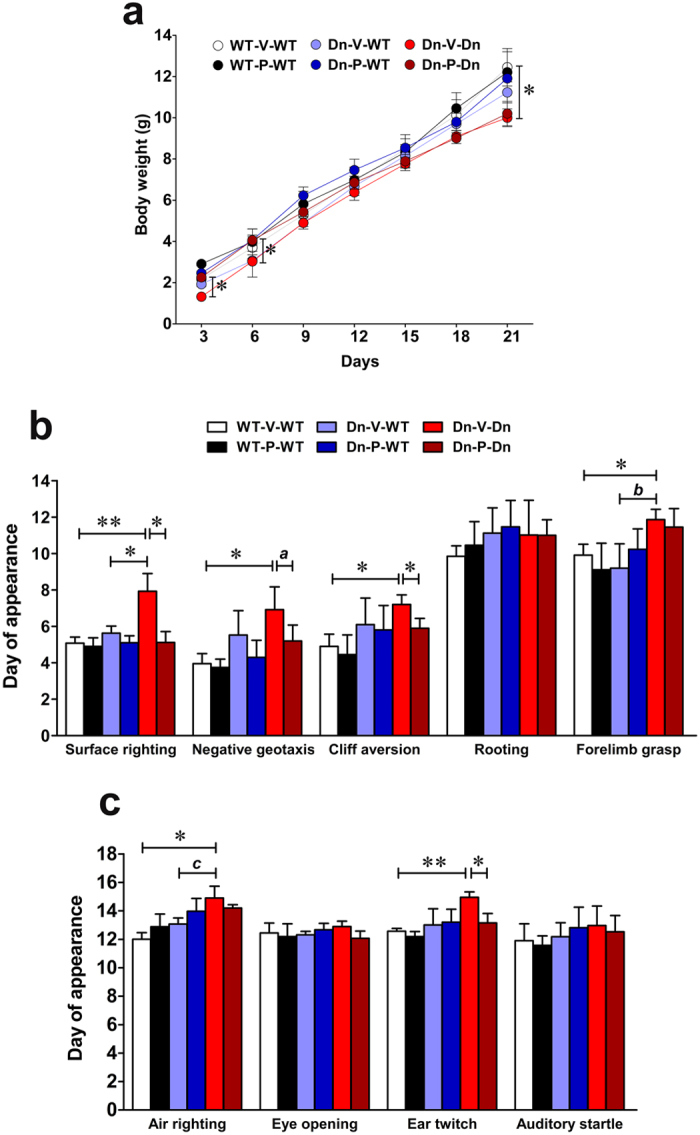
Prenatal to early postnatal treatment with P021 prevents delay in neurobehavioral development in Ts65Dn pups. (**a**) Body weight analysis of young mice from PND3-21. The Ts65Dn mice weighed less than WT mice on PND21. At early developmental stages (PND3 and PND6), P021 treatment induced significant weight gain in Ts65Dn mice, and brought it to WT level. (**b**,**c**) Neurobehavioral development evaluation of young mice from PND1-21. Ts65Dn mice exhibited significant delay in the development of surface righting, negative geotaxis, cliff aversion, forelimb grasp, air righting, and ear twitch. Prenatal to early postnatal treatment with P021 significantly prevented the delay in the appearance of surface righting, cliff aversion, and ear twitch. There was also a trend towards rescue of delay in the development of negative geotaxis. Data are presented as mean ± S.E.M. and are based on WT-V-WT (n = 11), WT-P-WT (n = 10), Dn-V-WT (n = 10), Dn-P-WT (n = 9), Dn-V-Dn (n = 11), and Dn-P-Dn (n = 12) mice. *p < 0.05; **p < 0.01; *a, p* = 0.09; *b, p* = 0.073; *c, p* = 0.072. One-way or two-way ANOVA with Bonferroni’s *post*-*hoc* test.

Developmental delay is nearly universal in DS^89^, and is also documented in Ts65Dn mice^18^,^21^. We assessed the effect of prenatal to early postnatal treatment with P021 on neurobehavioral development in Ts65Dn mice from PND1-21 (Fig. 2).

Overall, we found a significant group effect in body weight analysis [Fig. 2a; two-way ANOVA, *F*_(5,560)_ = 3.62, *p* = 0.0031] and a significant time effect [*F*_(6,560)_ = 152.13, *p* < 0.0001], however group × time interaction was not significant [*F*_(30,560)_ = 0.36, *p* = 0.995]. On PND21, the body weight of vehicle-treated Ts65Dn mice was significantly lower than vehicle-treated WT controls (Fig. 2a; WT-V-WT vs. Dn-V-Dn, *p* < 0.05). This agrees with previous studies which demonstrated lower body weight in Ts65Dn pups as compared to WT^22^,^90^. At early stages of development, P021 treatment induced weight gain in Ts65Dn mice (Fig. 2a; PND3 and PND6, Dn-V-Dn vs. Dn-P-Dn, *p* < 0.05). During the early developmental stages, mouse pups received P021 exclusively from mother’s milk, whereas at later stages pups also received P021 through directly consuming the chow. The weight gain during early developmental stages raises the possibility that P021 can be enriched in mother’s milk and/or exerts its maximal effect through breast feeding.

The Ts65Dn mice exhibited a delay in the achievement of certain developmental milestones. The differences were statistically significant for surface righting, negative geotaxis, cliff aversion, forelimb grasp, air righting, and ear twitch (Fig. 2b,c; WT-V-WT vs. Dn-V-Dn; *p* < 0.01 for surface righting and ear twitch, and *p* < 0.05 for negative geotaxis, cliff aversion, forelimb grasp, and air righting). There was no significant difference between the two control groups (Fig. 2b,c; WT-V-WT and Dn-V-WT) in any of the developmental milestone studied (*p* > 0.05). Overall, the Ts65Dn mice exhibited significant delay mainly in the achievement of developmental milestones which involve complex motor performance and skills^79^.

Prenatal to early postnatal treatment with P021 rescued the delay significantly in the development of surface righting, cliff aversion, and ear twitch (Fig. 2b,c; Dn-V-Dn vs. Dn-P-Dn, *p* < 0.05). We also observed a trend towards rescue of delay in the achievement of negative geotaxis (Fig. 2b,c; multiplicity adjusted Bonferroni’s *post hoc* test, *p* = 0.09).

### Prenatal to early postnatal treatment with P021 ameliorates pre-synaptic protein deficit, decreases GSK3β activity, and increases synaptic plasticity markers expression in 3-week-old Ts65Dn mice

**Figure 3:**
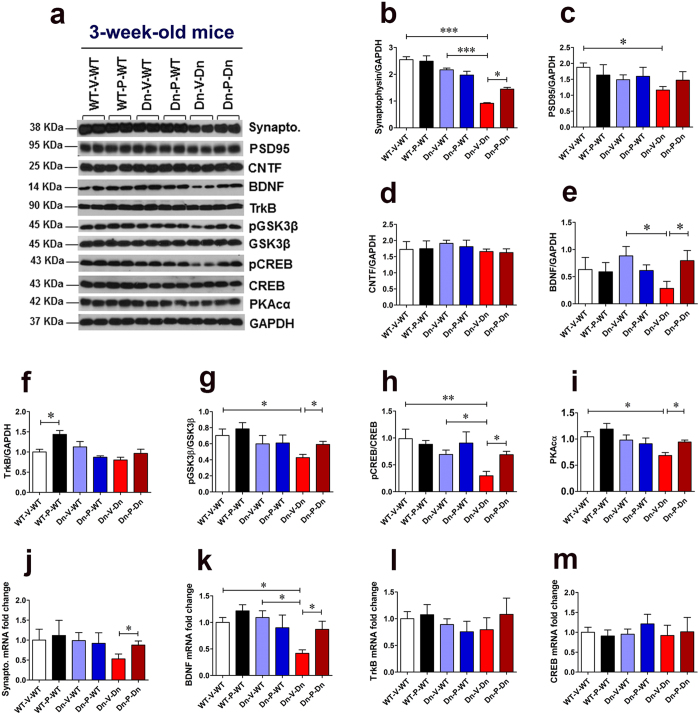
Prenatal to early postnatal treatment with P021 rescues synaptic deficit and increases expression of synaptic plasticity markers in 3-week-old Ts65Dn mice. (**a**–**i**) Western blot analyses of synaptophysin, PSD95, CNTF, BDNF, TrkB, pGSK3β (pSer9)/GSK3β, pCREB (pSer133)/CREB, and PKAcα in the hippocampus of 3-week-old mice. P021 treatment ameliorated deficits in synaptophysin, BDNF, and PKAcα expression, and increased pGSK3β/GSK3β and pCREB/CREB ratios. (**a**) Representative Western blots. (**b**–**i**) Quantification of the Western blots. (**j**–**m**) The mRNA levels of synaptophysin, BDNF, TrkB, and CREB in the cortex. P021 treatment significantly enhanced synaptophysin and BDNF mRNA levels. The data are presented as mean ± S.E.M. from WT-V-WT (n = 6), WT-P-WT (n = 7), Dn-V-WT (n = 7), Dn-P-WT (n = 7), Dn-V-Dn (n = 7), and Dn-P-Dn (n = 6) mice. *p < 0.05; **p < 0.01; ***p < 0.001. One-way ANOVA with Bonferroni’s *post*-*hoc* test.

The brains of DS individuals are known to exhibit several synaptic abnormalities at early developmental stages^91^,^92^,^93^,^94^. These synaptic abnormalities may be the major causative factor in developmental delay and early-onset AD-like learning and memory impairments in DS. Synaptophysin is a 38 KDa integral membrane glycoprotein located in pre-synaptic vesicles^95^. Previously, reduced synaptophysin levels were reported in the hippocampus of 8-day-old Ts65Dn mice^96^. We found a significantly reduced expression of synaptophysin in the hippocampus of 3-week-old Ts65Dn mice (Fig. 3a,b; WT-V-WT vs. Dn-V-Dn, *p* < 0.001; Dn-V-WT vss Dn-V-Dn, *p* < 0.001). Prenatal to early postnatal treatment with P021 ameliorated the synaptophysin deficit in Ts65Dn mice (Fig. 3a,b; Dn-V-Dn vs. Dn-P-Dn, *p* < 0.05). PSD95, a membrane-associated guanylate kinase, is the major scaffolding protein in the excitatory postsynaptic density (PSD) and a potent regulator of synaptic strength^97^,^98^. We found a significantly reduced PSD95 expression in the hippocampus of 3-week-old Ts65Dn mice (Fig. 3a,c; WT-V-WT vs. Dn-V-Dn, *p* < 0.05). There was no statistically significant effect of P021 treatment on PSD95 expression (Fig. 3a,c; Dn-V-Dn vs. Dn-P-Dn, *p* > 0.05). The CNTF expression level did not differ in the hippocampus of 3-week-old WT and Ts65Dn mice (Fig. 3a,d; WT-V-WT vs. Dn-V-Dn, *p* > 0.05). Prenatal to early postnatal treatment with P021 did not affect CNTF expression in Ts65Dn mice (Fig. 3a,d; Dn-V-Dn vs. Dn-P-Dn, *p* > 0.05).

BDNF is an important biological marker of synaptic plasticity. It plays a crucial role in both the early and late phases of LTP, the cellular substrate for learning and memory^99^,^100^,^101^,^102^. BDNF is also essential for basal level of hippocampal neurogenesis and for the survival and integration of new-born neurons into the hippocampal circuitry^103^,^104^. Previously, we showed that P021 exerts its beneficial effects on cognition via increasing BDNF expression^66^,^67^. Here, we found a significantly reduced expression of BDNF in the hippocampus of 3-week-old Ts65Dn mice (Fig. 3a,e; Dn-V-WT vs. Dn-V-Dn, *p* < 0.05). Prenatal to early postnatal treatment with P021 significantly ameliorated the deficit in BDNF expression (Fig. 3a,e; Dn-V-Dn vs. Dn-P-Dn, *p* < 0.05). As BDNF acts through TrkB receptor, we evaluated the expression levels of TrkB in the hippocampus. We did not find a significant difference in TrkB expression between 3-week-old WT and Ts65Dn mice (Fig. 3a,f; WT-V-WT vs. Dn-V-Dn, *p* > 0.05; Dn-V-WT vs. Dn-V-Dn, *p* > 0.05), and P021 treatment did not affect TrkB expression in Ts65Dn mice (Fig. 3a,f; Dn-V-Dn vs. Dn-P-Dn, *p* > 0.05). Nonetheless, P021 treatment significantly increased TrkB expression in WT mice (Fig. 3a,f; WT-V-WT vs. WT-P-WT, *p* < 0.05).

Previously, we showed that P021 reduces AD pathology via increased BDNF expression-mediated reduction in GSK3β activity^67^. GSK3β is one of the downstream effectors of BDNF/TrkB/PI3-kinase/AKT signal transduction pathway and can be inhibited by AKT-mediated phosphorylation on Ser9 (pGSK3β)^105^. APP-dependent alteration of GSK3β activity was shown to impair neurogenesis in Ts65Dn mice^106^. Drugs that increase GSK3β phosphorylation at Ser9, such as lithium and fluoxetine, have been shown to improve neurogenesis and cognition in Ts65Dn mice^22^,^23^,^106^,^107^. We thus evaluated the effect of prenatal to early postnatal treatment with P021 on phosphorylation of GSK3β at Ser9. We found a significantly reduced pGSK3β/GSK3β ratio, suggesting decreased phosphorylation of GSK3β at Ser9, in 3-week-old Ts65Dn mice as compared to WT controls (Fig. 3a,g; WT-V-WT vs. Dn-V-Dn, *p* < 0.05). P021 treatment significantly increased this inhibitory phosphorylation of GSK3β, thus decreasing its activity (Fig. 3a,g; Dn-V-Dn vs. Dn-P-Dn, *p* < 0.05).

We further quantified the expression level of a transcription factor known to play an evolutionarily critical role in long-term synaptic plasticity and memory formation: the phosphorylated form of CREB (Ser133)^108^. Trisomy 21 is reported to interfere with activation, i.e., phosphorylation of CREB^109^ which may be one of the mechanisms of early-onset neurocognitive dysfunction in DS^110^,^111^. We found a significantly reduced pCREB/CREB ratio in the hippocampus of 3-week-old Ts65Dn mice (Fig. 3a,h; WT-V-WT vs. Dn-V-Dn, *p* < 0.01; Dn-V-WT vs. Dn-V-Dn, *p* < 0.05). Prenatal to early postnatal treatment with P021 significantly ameliorated this deficit (Fig. 3a,h; Dn-V-Dn vs. Dn-P-Dn, *p* < 0.05).

Protein kinase A (PKA) is a major CREB kinase which activates it by phosphorylation at Ser133^112^,^113^. The holoenzyme of PKA is made up of a regulatory subunit dimer, and each regulatory subunit (R) is bound to a catalytic subunit (C). Under low levels of cAMP, the holoenzyme remains intact and is catalytically inactive. When the intracellular concentration of cAMP increases, cAMP binds to the two binding sites on each regulatory subunit, which results in the release of the catalytic subunits. Free catalytic subunits catalyze the phosphorylation of substrate proteins including CREB^114^. As we found a significantly reduced phosphorylation of CREB in 3-week-old Ts65Dn mice, we further quantified the PKA catalytic subunit α (PKAcα) levels in these mice. We found a significantly reduced PKAcα expression in 3-week-old Ts65Dn mice (Fig. 3a,i; WT-V-WT vs. Dn-V-Dn, *p* < 0.05). Prenatal to early postnatal treatment with P021 significantly increased PKAcα expression in these mice (Fig. 3a,i; Dn-V-Dn vs. Dn-P-Dn, *p* < 0.05).

To further assess the effect of prenatal to early postnatal P021 treatment on synaptophysin and BDNF in young Ts65Dn mice, we evaluated their messenger RNA (mRNA) levels in the cortex. We found that P021 treatment significantly increased synaptophysin mRNA level in 3-week-old Ts65Dn mice (Fig. 3j; Dn-V-Dn vs. Dn-P-Dn, *p* < 0.05). BDNF mRNA level was significantly reduced in 3-week-old Ts65Dn mice (Fig. 3k; WT-V-WT vs. Dn-V-Dn, *p* < 0.05; Dn-V-WT vs. Dn-V-Dn, *p* < 0.05), and P021 treatment significantly ameliorated this deficit (Fig. 3k; Dn-V-Dn vs. Dn-P-Dn, *p* < 0.05). We didn’t find any significant difference in TrkB and CREB mRNA levels among the study groups (Fig. 3l,m; one-way ANOVA, *p* = 0.7858 and *p* = 0.9441, respectively).

### Prenatal to early postnatal treatment with P021 ameliorates long-term memory deficit in adult life in Ts65Dn mice

**Figure 4:**
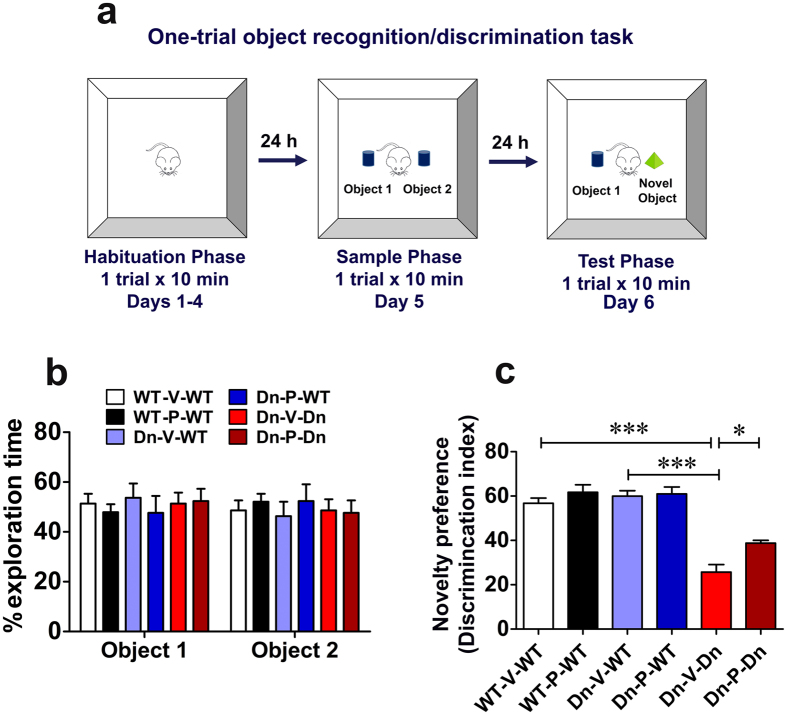
Prenatal to early postnatal treatment with P021 ameliorates impairment in long-term recognition memory in adult Ts65Dn mice. (**a**) The experimental design of one-trial object recognition/discrimination task for long-term memory. (**b**) Time spent exploring the objects during the sample phase of the object recognition/discrimination task. There was no difference among experimental groups. (**c**) In the test phase, the vehicle-treated Ts65Dn mice (Dn-V-Dn) exhibited a significantly impaired novelty preference (discrimination index, 24.7%) as compared to vehicle-treated WT mice (WT-V-WT, discrimination index, 56.8% and Dn-V-WT, discrimination index, 60%), suggesting a short-term memory deficit in these mice. The prenatal to early postnatal treatment with P021 ameliorated this deficit significantly; however, these mice (Dn-P-Dn, discrimination index, 38.8%) still did not reach the WT level of novelty preference. Data are presented as mean ± S.E.M. and are based on WT-V-WT (n = 10), WT-P-WT (n = 12), Dn-V-WT (n = 12), Dn-P-WT (n = 11), Dn-V-Dn (n = 13), and Dn-P-Dn (n = 12) mice. *p < 0.05; ***p < 0.001. One-way ANOVA with Bonferroni’s *post*-*hoc* test.

We evaluated long-term declarative memory using one-trial object recognition/discrimination task, with a retention period of 24 h (Fig. 4a). The medial temporal lobe structures including the hippocampus and perirhinal, entorhinal, parahippocampal cortices govern the appropriate encoding, consolidation, and retrieval of information related to discrete events^115^,^116^. This process ultimately leads to recognition memory, which consists of two components: (1) familiarity, a global awareness of the features of a stimulus, and (2) recollection, knowledge of the stimulus in the context of other information, such as what, where, or under which context the stimulus was encountered^115^,^116^,^117^,^118^. The episodic or recollection component of the recognition memory is thus dependent on the integrated conceptualization of “what”, “where”, and “which” parameters of a situation. In the one-trial object recognition/discrimination task for long-term memory, animals explore two different objects which they have to identify as either novel or familiar based on the memory of an earlier experience with one of the two objects which they were exposed to in the same open-field arena^72^. The animals are expected to explore the familiar object for a shorter time than the novel object because the representation of the former is still available in memory^72^.

Impairment in visual object long-term memory in DS individuals is well documented (for review, ref. 119). Long-term memory deficit using novel object recognition task has also been demonstrated in Ts65Dn mice^32^,^35^. We thus assessed the effect of prenatal to early postnatal treatment with P021 on object recognition long-term memory in adult (5-7-month-old) Ts65Dn mice. All groups of mice, on average, spent equal time exploring the objects during the sample phase (Fig. 4b; ANOVA, *p* = 0.7567). Test phase was performed 24 h after the sample acquisition phase, and showed a severe impairment in vehicle-treated Ts65Dn mice. Indeed, the discrimination index for the Dn-V-Dn group was 25.7 ± 3.5% compared to 56.8 ± 2.3% of the WT-V-WT group (Fig. 4c; *p* < 0.001). Similarly, the discrimination index for Dn-V-WT (60 ± 2.5%) was significantly higher than that of Dn-V-Dn group (Fig. 4c; *p* < 0.001). These data indicated a severe deficit of long-term memory in Ts65Dn mice. Prenatal to early postnatal treatment with P021 significantly improved the performance of Ts65Dn mice. The discrimination index in Dn-P-Dn versus Dn-V-Dn group was 38.8 ± 1.2 versus 25.7 ± 3.5% (Fig. 4c; *p* < 0.05). Nonetheless, the Dn-P-Dn group was still significantly impaired compared to WT-V-WT and Dn-V-WT control groups (*p* < 0.001). Thus, the rescue of long-term memory with prenatal to early postnatal treatment with P021 was only partial. The performance of WT mice was not affected by P021 treatment (Fig. 4c; WT-V-WT vs. WT-P-WT, *p* > 0.05; Dn-V-WT vs. Dn-P-WT, *p* > 0.05).

### Prenatal to early postnatal treatment with P021 attenuates spatial reference learning and memory deficit in adult life in Ts65Dn mice

**Figure 5:**
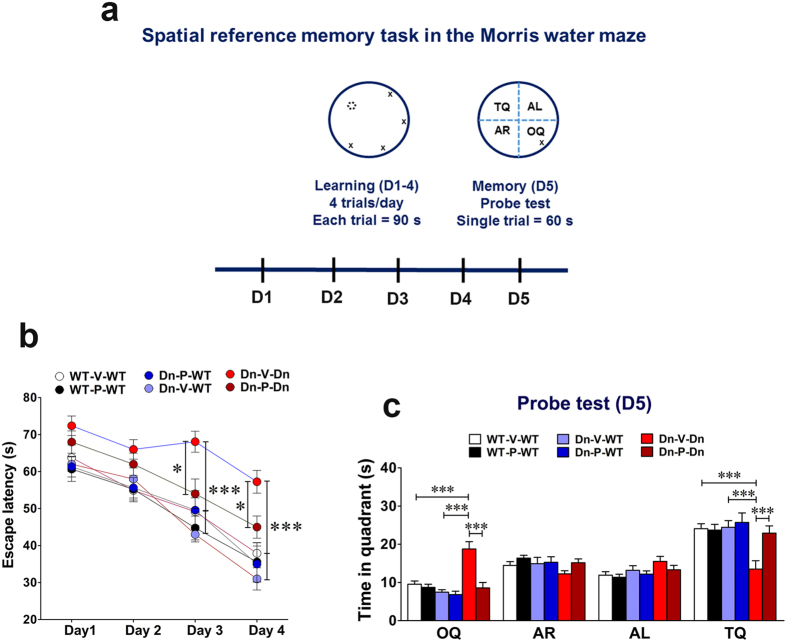
Prenatal to early postnatal treatment with P021 rescues spatial reference learning and memory impairment in adult Ts65Dn mice. (**a**) The experimental paradigm of the spatial reference memory task in Morris water maze. (**b**) The escape latency during the training phase (D1-4). Compared to WT controls, the Ts65Dn mice exhibited significant delay in finding the escape platform which was ameliorated by P021 treatment. (**c**) The time spent in each quadrant in probe trial. The Ts65Dn mice spent significantly less time in the target quadrant as compared to WT mice. P021 treatment rescued this memory deficit in Ts65Dn mice. Data are presented as mean ± S.E.M. and are based on WT-V-WT (n = 10), WT-P-WT (n = 12), Dn-V-WT (n = 12), Dn-P-WT (n = 11), Dn-V-Dn (n = 13), and Dn-P-Dn (n = 12) mice. *p < 0.05; ***p < 0.001. Two-way ANOVA with Bonferroni’s *post*-*hoc* test. TQ, target quadrant; AR, adjacent right quadrant; AL, adjacent left quadrant, OQ, opposite quadrant.

We assessed spatial reference learning and memory abilities in the MWM task (Fig. 5a), a cognitive paradigm in which Ts65Dn mice are well documented to be severely impaired^28^,^29^,^30^,^31^,^32^,^33^,^34^,^36^. The MWM task evaluates hippocampus-dependent reference learning and memory in rodents, and involves the use of a spatial navigational strategy to locate a fixed submerged escape platform. The hippocampal formation formulates a spatial map based on distal environmental cues^86^,^120^, and plays an essential role in memory storage, consolidation, and retrieval of the spatial information^86^,^121^,^122^.

We found that the latency to locate the submerged platform on the 3^rd^ and 4^th^ days of training were significantly longer in Dn-V-Dn group as compared to the control groups, WT-V-WT and Dn-V-WT (Fig. 5b; *p* < 0.001) suggesting impairment of spatial reference learning and memory in adult Ts65Dn mice. Prenatal to early postnatal treatment with P021 significantly rescued this spatial learning and memory deficit in Ts65Dn mice (Fig. 5b; 3^rd^ and 4^th^ days of training, Dn-V-Dn vs. Dn-P-Dn, *p* < 0.05). The probe trial on the 5^th^ day confirmed the spatial memory impairment of Ts65Dn mice: the WT-V-WT and Dn-V-WT groups spent significantly longer time in the TQ (where the platform was located during the training trials); in contrast, Dn-V-Dn mice did not exhibit any preference for the TQ (Fig. 5c; *p* < 0.001). Here too, the prenatal to early postnatal treatment with P021 completely rescued the cognitive dysfunction shown by Ts65Dn mice (Fig. 5c; Dn-V-Dn vs. Dn-P-Dn, *p* < 0.001). Additionally, Dn-V-Dn mice spent significantly greater time in OQ (Fig. 5c; WT-V-WT vs. Dn-V-Dn, *p* < 0.001; Dn-V-WT vs. Dn-V-Dn, *p* < 0.001) which was corrected by P021 treatment (Fig. 5c; Dn-V-Dn vs. Dn-P-Dn, *p* < 0.001).

### Prenatal to early postnatal treatment with P021 rescues pre-synaptic protein deficit, decreases GSK3β activity, and increases synaptic plasticity markers expression in adult Ts65Dn mice

**Figure 6:**
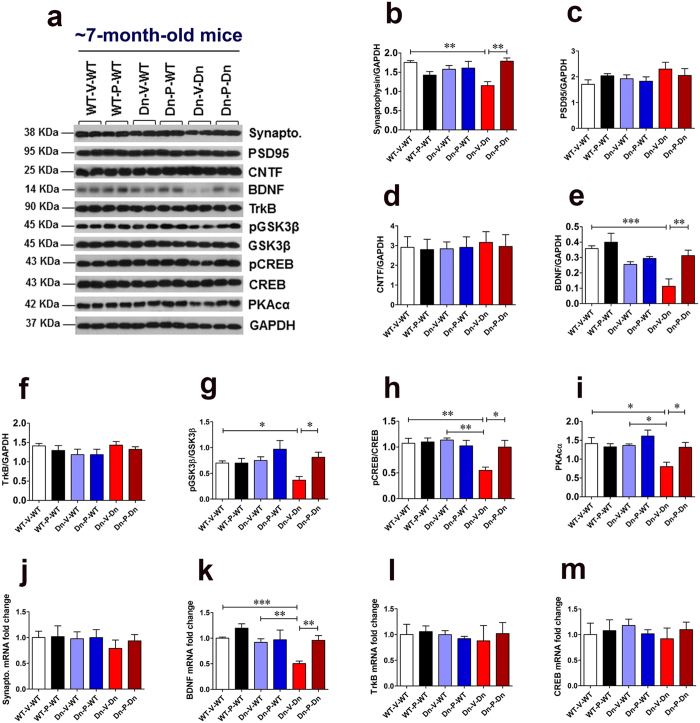
Prenatal to early postnatal treatment with P021 restores expression of synaptic density and synaptic plasticity markers in adult Ts65Dn mice. (**a**–**i**) Western blot analyses of synaptophysin, PSD95, CNTF, BDNF, TrkB, pGSK3β (pSer9)/GSK3β, pCREB(pSer133)/CREB, and PKAcα in the hippocampus of ~7-month-old mice. P021 treatment ameliorated deficits in synaptophysin, BDNF, and PKAcα expression, and increased pGSK3β/GSK3β and pCREB/CREB ratios. (**a**) Representative Western blots. (**b**–**i**) Quantification of the Western blots. (**j**–**m**) The mRNA levels of synaptophysin, BDNF, TrkB, and CREB in the cortex. P021 treatment ameliorated BDNF mRNA level deficit. The data are presented as mean ± S.E.M. from WT-V-WT (n = 7), WT-P-WT (n = 7), Dn-V-WT (n = 6), Dn-P-WT (n = 6), Dn-V-Dn (n = 7), and Dn-P-Dn (n = 7) mice. *p < 0.05; **p < 0.01; ***p < 0.001. One-way ANOVA with Bonferroni’s *post*-*hoc* test.

To evaluate the cellular and molecular basis of beneficial effects of prenatal to early postnatal treatment with P021 on learning and memory, we evaluated the synaptophysin, PSD95, BDNF, pGSK3β/GSK3β, pCREB/CREB, and PKAcα expression levels. Like young mice, we found a significant deficit in the pre-synaptic protein, synaptophysin, expression in adult (~7-month-old) Ts65Dn mice (Fig. 6a,b; WT-V-WT vs. Dn-V-Dn, *p* < 0.01). P021 treatment restored the synaptophysin levels to that of WT mice (Fig. 6a,b; Dn-V-Dn vs. Dn-P-Dn, *p* < 0.01; WT-V-WT vs. Dn-P-Dn, *p* > 0.05). We didn’t find any statistically significant difference in post-synaptic protein, PSD95, expression among study groups (Fig. 6a,c; ANOVA, *p* = 0.5734). Nonetheless, we found a trend towards increased PSD95 expression in ~7-month-old Ts65Dn mice as compared to WT controls (Fig. 6a,c; WT-V-WT vs. Dn-V-Dn, multiplicity adjusted Bonferroni’s *post hoc* test, *p* = 0.095). In congruence with our data, previously, increased PSD95 expression has been documented in the hippocampus of 5-month-old Ts65Dn mice^123^. The CNTF expression didn’t differ significantly between study groups (Fig. 6a,d; ANOVA, *p* = 0.5329).

Similar to young mice, we found that prenatal to early postnatal treatment with P021 ameliorated the deficit in BDNF expression in the hippocampus of ~7-month-old Ts65Dn mice (Fig. 6a,e; WT-V-WT vs. Dn-V-Dn, *p* < 0.001; Dn-V-Dn vs. Dn-P-Dn, *p* < 0.01). Like young mice, we didn’t find a significant difference in TrkB expression levels among different groups of adult mice (Fig. 6a,f; one-way ANOVA, *p* = 0.4868). The pGSK3β/GSK3β ratio was significantly reduced in ~7-month-old Ts65Dn mice (Fig. 6a,g; WT-V-WT vs. Dn-V-Dn, *p* < 0.05). Prenatal to early postnatal treatment with P021 increased the inhibitory phosphorylation of GSK3β, Ser9 (Fig. 6a,g; Dn-V-Dn vs. Dn-P-Dn, *p* < 0.05) suggesting a possible mechanism by which this treatment could have exerted its beneficial effect on cognition in Ts65Dn mice.

As CREB phosphorylation is required to induce BDNF expression, and pCREB (Ser133) is a molecular marker of long-term memory, we evaluated the pCREB/CREB ratio in adult mice. We found a significantly reduced pCREB/CREB ratio in ~7-month-old Ts65Dn mice as compared to WT controls (Fig. 6a,h; WT-V-WT vs. Dn-V-Dn, *p* < 0.01; Dn-V-WT vs. Dn-V-Dn, *p* < 0.01). P021 treatment significantly increased the phosphorylation of CREB levels (Fig. 6a,h; Dn-V-Dn vs. Dn-P-Dn, *p* < 0.05). Correspondingly, PKAcα expression was significantly reduced in ~7-month-old Ts65Dn mice (Fig. 6a,i; WT-V-WT vs. Dn-V-Dn, *p* < 0.05; Dn-V-WT vs. Dn-V-Dn, *p* < 0.05). P021 treatment significantly increased PKAcα expression in these mice (Fig. 6a,i; Dn-V-Dn vs. Dn-P-Dn, *p* < 0.05).

The RT-qPCR analysis didn’t reveal a significant difference in synaptophysin mRNA levels among study groups (Fig. 6j; one-way ANOVA, *p* = 0.5642). However, in adult (~7-month-old) Ts65Dn mice, there was a significant deficit in BDNF mRNA levels which was rescued by P021 treatment (Fig. 6k; WT-V-WT vs. Dn-V-Dn, *p* < 0.001; Dn-V-WT vs. Dn-V-Dn, *p* < 0.01; Dn-V-Dn vs. Dn-P-Dn, *p* < 0.01). The TrkB and CREB mRNA levels didn’t differ among study groups (Fig. 6l,m; one-way ANOVA, *p* = 0.7869 and *p* = 0.8934, respectively).

### Prenatal to early postnatal treatment with P021 does not affect body weight and anxiety-like behavior in adult Ts65Dn mice

Prenatal to early postnatal treatment with P021 in the present study did not affect the general behavioral profile of the adult mice as it neither amplified nor diminished alterations of general behavior induced by trisomy. Besides P021 treatment did not produce any new changes in general behavior. The pregnant mothers which received P021 treatment via diet also did not show any treatment-induced alteration in their general physical condition, as evaluated up to 2–3 months after weaning the pups.

**Figure 7:**
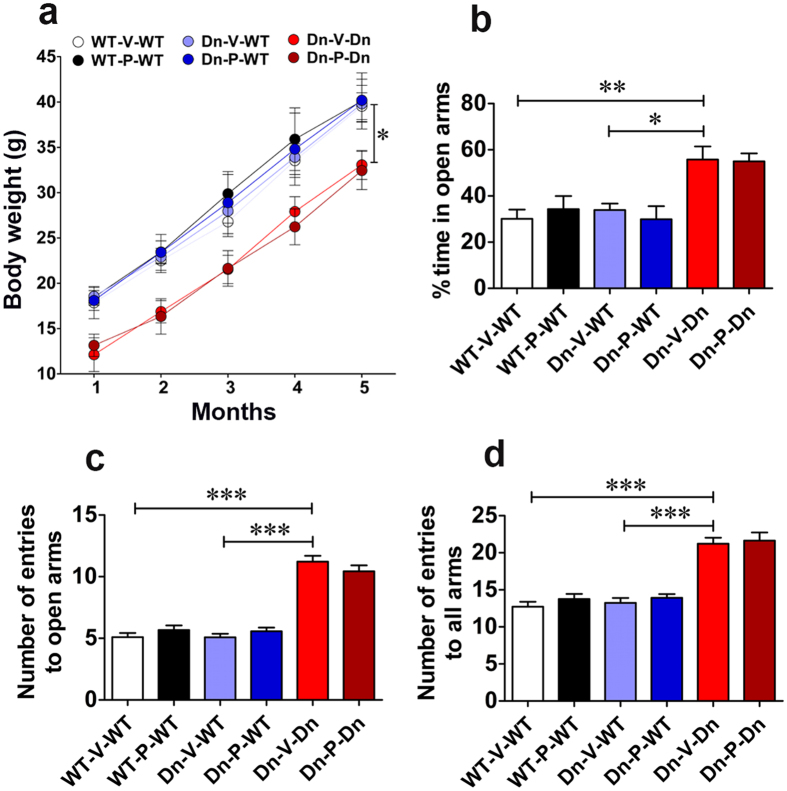
Prenatal to early postnatal treatment with P021 does not affect body weight and anxiety-like behavior in adult Ts65Dn mice. (**a**) The monthly evaluation of body weight of study mice. The Ts65Dn mice weighed less than WT controls. No effect of P021 on body weight was observed. (**b–d**) Evaluation of anxiety-like behavior by measuring percentage of time spent in open arm (**b**), number of entries in open arm (**c**), and number of entries to all arms (**d**), in a 15 min-trial in elevated plus maze. Ts65Dn mice exhibited hyperactivity in the elevated plus maze. No effect of P021 treatment was observed. Data are presented as mean ± S.E.M. and are based on WT-V-WT (n = 10), WT-P-WT (n = 12), Dn-V-WT (n = 12), Dn-P-WT (n = 11), Dn-V-Dn (n = 13), and Dn-P-Dn (n = 12) mice. *p < 0.05; **p < 0.01***p < 0.001. One-way or two-way ANOVA with Bonferroni’s *post*-*hoc* test.

The administration of the full-length human recombinant CNTF protein in clinical trials has been reported to cause anorexia, skeletal muscle loss, hyperalgesia, severe cramps, and muscle pain^60^. Thus, we performed a monthly evaluation of body weight of mice in longitudinal study to assess if P021 treatment induced any changes. Overall, we observed a significant group effect in body weight [Fig. 7a; 2-way ANOVA, *F*_(5,410)_ = 14.28, *p* < 0.0001] and its change with age [*F*_(4,410)_ = 100.25, *p* < 0.0001], however, group × age interaction was not significant [*F*_(20,410)_ = 0.10, *p* = 1.0]. Bonferroni’s *post hoc* analysis revealed a significant difference between WT-V-WT and Dn-V-Dn groups at all ages at which body weight was evaluated (Fig. 7a; *p* < 0.05). Similarly, a statistically significant difference was found between the other control group, Dn-V-WT, and the Dn-V-Dn group (Fig. 7a; *p* < 0.05). These data showed that Ts65Dn mice weighed less than their WT counterparts, as is reported in literature^29^. Prenatal to early postnatal treatment with P021 did not induce any significant changes in body weight in adult Ts65Dn mice (Fig. 7a; Dn-V-Dn vs. Dn-P-Dn, *p* > 0.05). Also, this treatment did not produce any alterations in body weight of WT mice (Fig. 7a; WT-V-WT vs. WT-P-WT, *p* > 0.05; Dn-V-WT vs. Dn-P-WT, *p* > 0.05).

Hyperactivity and decreased level of anxiety-like behavior are reported to occur in the elevated plus maze task in Ts65Dn mice as compared to WT controls^124^,^125^,^126^. We found that the Ts65Dn mice spent a significantly higher amount of time in open arms as compared to the control mice (Fig. 7b; WT-V-WT vs. Dn-V-Dn, *p* < 0.01; Dn-V-WT versus Dn-V-Dn, *p* < 0.05). In rodents, open arms are more anxiogenic than closed arms. These data showed that Ts65Dn mice are less anxious as they did not have same aversion to open arms as control mice. Nonetheless, the Ts65Dn mice had not only a higher number of open arm entries but also a higher number of entries to all arms (Fig. 7c,d; WT-V-WT vs. Dn-V-Dn, *p* < 0.001; and Dn-V-WT vs. Dn-V-Dn, *p* < 0.001 for both entries to open arms and all arms). These data indicate that Ts65Dn mice are hyperactive in the elevated plus maze as compared to WT controls, and this hyperactivity may have masked the actual evaluation of anxiety-like behavior in the elevated plus maze. We did not find any significant effect of prenatal to early postnatal treatment with P021 on anxiety-like behavior and activity level in Ts65Dn mice in elevated plus maze task (Fig. 7b–d; Dn-V-Dn vs. Dn-P-Dn, *p* > 0.05).

### Prenatal to early postnatal treatment with P021 reduces elevated spontaneous locomotor activity in adult Ts65Dn mice

**Figure 8:**
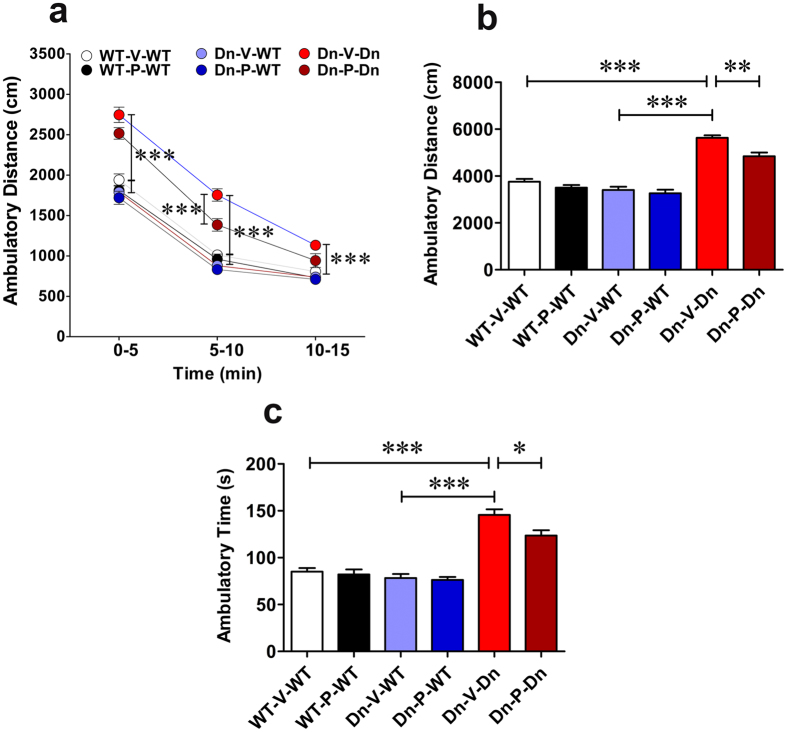
Prenatal to early postnatal treatment with P021 decreases spontaneous locomotor activity in adult Ts65Dn mice. (**a**–**c**) Ambulatory distance and time during 15 min-trials in the open field free exploration task. The locomotor activity was significantly increased in Ts65Dn mice as seen from increased ambulatory distance and time. Prenatal to early postnatal treatment with P021 significantly reduced these locomotion parameters. Data are presented as mean ± S.E.M. and are based on WT-V-WT (n = 10), WT-P-WT (n = 12), Dn-V-WT (n = 12), Dn-P-WT (n = 11), Dn-V-Dn (n = 13), and Dn-P-Dn (n = 12) mice. *p < 0.05; **p < 0.01***p < 0.001. One-way or two-way ANOVA with Bonferroni’s *post*-*hoc* test.

Elevated spontaneous locomotor activity was reported extensively in the Ts65Dn mice^18^,^31^,^33^,^35^,^127^,^128^. We analyzed the exploratory behavior and general locomotor activity in an open field arena. We observed a significantly greater locomotor activity in Ts65Dn mice as compared to control mice (Fig. 8). The ambulatory distance as analyzed in 3 intervals of 5 min each was significantly higher in Ts65Dn mice (Fig. 8a; 2-way ANOVA, *F*_(5,246)_ = 67.04, *p* < 0.0001; WT-V-WT vs. Dn-V-Dn, *p* < 0.001; Dn-V-WT vs. Dn-V-Dn, *p* < 0.001). The total ambulatory distance over the 15-min trial was also significantly greater in Ts65Dn mice versus control groups (Fig. 8b; WT-V-WT vs. Dn-V-Dn, *p* < 0.001; Dn-V-WT vs. Dn-V-Dn, *p* < 0.001). Prenatal to early postnatal treatment with P021 significantly reduced the locomotor activity in Ts65Dn mice (Fig. 8a; 5–10 min interval, Dn-V-Dn vs. Dn-P-Dn, *p* < 0.001; Fig. 8b; total 15 min trial, Dn-V-Dn vs. Dn-P-Dn, *p* < 0.01). The ambulatory time was also greater in Ts65Dn mice than WT controls (Fig. 8c; WT-V-WT vs. Dn-V-Dn, Dn-V-WT, *p* < 0.001; Dn-V-WT vs. Dn-V-Dn, *p* < 0.001). Prenatal to early postnatal treatment with P021 significantly reduced the ambulatory time in Ts65Dn mice (Fig. 8c; Dn-V-Dn vs. Dn-P-Dn, *p* < 0.05). The ambulatory distance and time in WT mice were not affected by P021 treatment (Fig. 8a–c; *p* > 0.05). Thus, prenatal to early postnatal treatment with P021 selectively altered the exploratory behavior, decreasing aberrantly elevated spontaneous locomotor activity in Ts65Dn mice.

## Discussion

The present study provides novel evidence that embryonic and early postnatal treatment with a neurotrophic factor small-molecule peptide mimetic can not only ameliorate developmental delay but also AD-like learning and memory impairments in adult life in a mouse model of DS. These data suggest that adjusting the brain milieu by providing neurotrophic support at critical period of brain development can be an effective therapeutic strategy for developmental disability and AD in DS.

In recent years, several pharmacological agents have been used in prenatal and/or early postnatal treatment studies with beneficial outcomes in DS mouse models. These agents include active peptide fragments of activity-dependent neuroprotective protein (ADNP) and activity-dependent neurotrophic factor (ADNF), NAP + SAL^21^,^34^, antioxidant, α-tocopherol (vitamin E)^129^, dietary supplement, choline^24^,^130^,^131^,^132^, antidepressant with neurogenic effect, fluoxetine^22^,^133^,^134^,^135^,^136^, synthetic activator of Sonic hedgehog (Shh) pathway, SAG^30^, and major polyphenol of green tea and dual-specificity tyrosine phosphorylation-regulated kinase 1A (DYRK1A) inhibitor, epigallocatechin-3-gallate (EGCG)^137^. To the best of our knowledge, the present study is the first employing a CNTF- or BDNF-based prenatal to early postnatal pharmacotherapy in a DS mouse model which showed beneficial effect both on neurodevelopment and on cognition in adult life.

P021 is a small water-soluble compound that was successfully administered orally in the present study. P021 has suitable pharmacokinetic properties for oral administration, is BBB permeable, and does not induce the systemic adverse effects of recombinant CNTF or BDNF^66^,^67^. P021 was shown to rescue cognitive impairment in rodent models of AD via increased BDNF expression^66^,^67^. In the present study, P021 administered orally at nanomolar level during embryonic and early postnatal period reached significant amounts in offsprings’ brains to exert beneficial effect on neurobehavioral development and cognition, probably via increased BDNF and pCREB expression, reduction in GSK3β activity, and by rescue of the synaptic deficit. Unlike recombinant CNTF, which has been shown to cause anorexia, skeletal muscle loss, hyperalgesia, severe cramps, and muscle pain in humans^60^, in the present study, we did not find any alterations in body weight and general physical state in P021-treated mice.

In the newborn period, DS infants exhibit delay in achievement of developmental motor and sensory milestones, as well as in olfactory, auditory, and visual sensitivity^94^,^138^,^139^,^140^. Our data are congruent with previous studies that have demonstrated delay in the acquisition of developmental milestones during the early postnatal period in Ts65Dn mice^18^,^21^,^141^. Like DS infants, the Ts65Dn mice ultimately achieve developmental milestones but with significant delay as compared to their WT littermates. The progression of infant motor and sensory development has previously been linked to cognition and motor skills in adulthood in humans^142^,^143^,^144^. A recent study reported a relationship between early motor skills and executive function in DS^145^. Similarly, in Ts65Dn mice, a positive correlation was recently reported between acquisition of developmental milestones during early postnatal period and cognitive performance in adult life^141^. The present study showed that prenatal to early postnatal treatment with P021 rescues the delay in some of the motor and sensory developmental milestones. These data suggest that providing neurotrophic support during fetal and early postnatal period can compensate for the developmental delay in Ts65Dn mice, and may result in improved cognitive performance in adult life. It was not possible for us to do a correlation analysis between acquisition of developmental milestones during the first 3 weeks of life and cognitive performance at adult stage as the data were collected from two separate batches of mice. Nonetheless, based on recent literature on Ts65Dn mice^141^, we speculate that at least one of the contributory factor in improved learning and memory performance in adult life in P021 treated Ts65Dn mice might be the amelioration of developmental delay during early postnatal period.

By the age 40, almost all DS individuals develop AD histopathological hallmarks, and by age 60, ~70% develop AD-like cognitive dysfunction^2^. The DS individuals show deficits in spatial and hippocampus-dependent learning paradigms^146^. The Ts65Dn mice exhibit similar behavioral deficits in spatial and hippocampus dependent learning paradigms such as the Morris water maze and novel object recognition/discrimination tasks^28^,^29^,^30^,^31^,^32^,^33^,^34^,^36^. In the spatial reference memory task, the hippocampus is crucial for processing information on relationships among distal environmental cues into a spatial map, and is also essential for memory storage, consolidation, and restitution of the spatial information^86^,^120^,^121^,^122^. We found that in adult Ts65Dn mice, the learning and memory performance was severely impaired compared to WT controls. These data show that Ts65Dn mice were not able to encode, store, and/or recognize spatial representation of the environment and co-ordinates of the submerged platform. Prenatal to early postnatal treatment with P021 ameliorated these deficits. We found that P021 treatment during early stages of brain development improved not only the spatial learning but also the spatial memory as the performance of P021-treated Ts65Dn mice was comparable to that of WT controls in probe trial. These results suggest that the P021 treatment enhanced memory consolidation mechanisms in Ts65Dn mice.

Impairment of hippocampus-dependent long-term memory, a major hallmark of AD, is also found in DS^147^. We employed one-trial object recognition/discrimination task to assess hippocampus-dependent long-term declarative memory. Impairment in this task is well documented in Ts65Dn mice^32^,^35^. The present study showed profound impairment of long-term memory in Ts65Dn mice. The Ts65Dn mice showed a lack of preference for the novel object most probably because of the failure of the encoding, storage, and retrieval of the memory related to familiar object representation. Prenatal to early postnatal treatment with P021 significantly ameliorated this long-term memory impairment in Ts65Dn mice.

Synaptic plasticity, the property of synapses to undergo long-term changes in synaptic strength, is thought to be the cellular substrate of learning and memory^148^. It has been suggested that AD-like cognitive deficits in Ts65Dn mice are strongly associated with failure of hippocampal LTP^26^, aberrant expression of synaptic proteins in the hippocampus^149^, and structural alterations in dendritic spines in the hippocampus and the cortex^150^. The developing DS brain also exhibits markedly aberrant synaptogenesis and dendritic hypotrophy^93^,^151^. In the present study, we found a significant deficit in the expression of the pre-synaptic protein, synaptophysin, in the hippocampus of both young and old Ts65Dn mice. Prenatal to early postnatal treatment with P021 rescued this deficit not only in young mice but also in adult mice. These data provide strong evidence that early neurotrophic treatment can exert long-lasting effect on pre-synaptic loss in Ts65Dn mice. As pre-synaptic vesicle proteins like synaptophysin are known to play an essential role in synaptic plasticity^152^, the increase in synaptophysin expression in P021-treated Ts65Dn mice might have contributed to their better cognitive performance.

BDNF is one of the most potent modulators of synaptic plasticity, and plays a crucial role in both the early and late phases of LTP^99^,^100^,^101^,^102^. It is also essential for hippocampal neurogenesis, and for the survival and integration of new-born neurons into the hippocampal circuitry^103^,^104^. Reduced expression of BDNF has been reported in the hippocampus^153^ and the cerebral cortex^154^ of DS fetuses. Young Ts65Dn mice also have reduced BDNF levels in the hippocampus^22^. Similarly, adult Ts65Dn mice were shown to have reduced BDNF expression in the frontal cortex^155^. In the present study, we found reduced BDNF expression and mRNA levels in the brains of both young and adult Ts65Dn mice. This deficit was ameliorated by prenatal to early postnatal P021 treatment. Previously, we showed that P021 exerts its neurogenic and neurotrophic effects in rodent model of AD by increasing BDNF transcription and its expression^66^,^67^. The results of the present study further strengthen this hypothesis. As compared to the previous studies in AD models^66^,^67^, in the present study, P021 was only administered during the early developmental stages in Ts65Dn mice. Remarkably, this treatment not only increased BDNF transcription and its expression in young mice but the effect persisted until adulthood. This data also support the recently proposed hypothesis that treatment during early brain development can have long-lasting effects in DS mouse models^41^,^42^.

The transcription of BDNF gene is regulated by CREB phosphorylation^156^. The phosphorylation of CREB is known to play a critical role in long-term synaptic plasticity and memory formation^108^. Furthermore, it triggers the transcription of c-fos and zif268 which both contribute to memory consolidation^157^. We observed significantly less pCREB and PKAcα (major CREB kinase catalytic subunit) expression in both young and adult Ts65Dn mice. This deficit was rescued by prenatal to early postnatal P021 treatment, hinting to another pathway by which this treatment might have ameliorated learning and memory deficits in Ts65Dn mice.

Another possible mechanism by which P021 could have exerted its beneficial effects in Ts65Dn mice is reduction in GSK3β activity. Previously, APP-dependent alteration of GSK3β activity was demonstrated to cause impairment in neurogenesis in Ts65Dn mice^106^; and GSK3β inhibitors improved neurogenesis and memory in Ts65Dn mice^22^,^23^,^106^,^107^. P021 was previously documented to be a GSK3β inhibitor via BDNF/TrkB/PI3-kinase/AKT pathway^67^. In the present study, we also found that prenatal to early postnatal treatment with P021 significantly reduced GSK3β activity by increasing its inhibitory phosphorylation at Ser9, both in young and adult Ts65Dn mice. This reduction in GSK3β activity may have contributed significantly to the beneficial effect of P021 on developmental delay in new-born period and AD-like memory deficit in adult life in Ts65Dn mice.

Elevated spontaneous locomotor activity has been documented both in young and old Ts65Dn mice^18^,^31^,^33^,^35^,^127^,^128^. Also in DS individuals, hyperkinetic and hyperactivity disorders are common^158^,^159^. Aberrant motor behavior and disinhibition are also reported in AD patients^160^,^161^. We found enhanced spontaneous locomotor activity in adult Ts65Dn mice which was significantly ameliorated by prenatal to early postnatal P021 treatment. These data suggest that early treatment can not only rescue cognitive impairment but also benefit other behavioral disturbances in a DS mouse model.

In summary, we found that prenatal to early postnatal treatment with a neurotrophic factor small-molecule peptide mimetic in Ts65Dn mice rescues developmental delay and AD-like learning and memory impairment, probably via increased BDNF and pCREB expression, reduced GSK3β activity, and amelioration of pre-synaptic protein deficit. The present study further corroborates the recent hypothesis that trisomy 21-linked brain abnormalities can be prevented with embryonic and neonatal treatment. Furthermore, we show a novel therapeutic modality, i.e., neurotrophic factor small-molecule mimetic with potent neurogenic and neurotrophic effects, that can robustly prevent the AD-like cognitive impairment in a DS mouse model without exerting any significant adverse effect.

## Additional Information

**How to cite this article:** Kazim, S. F. *et al*. Early neurotrophic pharmacotherapy rescues developmental delay and Alzheimer’s-like memory deficits in the Ts65Dn mouse model of Down syndrome. *Sci. Rep.* **7**, 45561; doi: 10.1038/srep45561 (2017).

**Publisher's note:** Springer Nature remains neutral with regard to jurisdictional claims in published maps and institutional affiliations.
